# Whether the risk of gestational diabetes mellitus is affected by *TNF-α*, *IL-6*, *IL-10* or *ADIPOQ* polymorphisms: a meta-analysis

**DOI:** 10.1186/s13098-020-00582-8

**Published:** 2020-09-17

**Authors:** Qiqi Huang, Yi Wang, Binbin Gu, Yanwen Xu

**Affiliations:** 1 Department of Nutrition, The First Affiliated Hospital of Wenzhou Medical University, Wenzhou, 325000 China; 2 Department of Endocrinology, Huzhou Hospital of Traditional Chinese Medicine, Zhejiang University of Traditional Chinese Medicine, No.315 of South Chaoyang Street, Huzhou, 313000 China

**Keywords:** Gestational diabetes mellitus (GDM), Tumor necrosis factor-α (*TNF-α*), Interleukin-6 (*IL-6*), Interleukin-10 (*IL-10*), Adiponectin (ADIPOQ)

## Abstract

**Background:**

Whether polymorphisms in tumor necrosis factor-α (*TNF-α*), interleukin-6 (*IL-6*), interleukin-10 (*IL-10*) or adiponectin (*ADIPOQ*) influence the risk of gestational diabetes mellitus (GDM) or not remain inconclusive. Therefore, the authors conducted a meta-analysis to robustly assess relationships between polymorphisms in *TNF-α*, *IL-6*, *IL-10* or *ADIPOQ* and the risk of GDM by merging the results of eligible publications.

**Methods:**

A through literature searching in Medline, Embase, Wanfang, VIP and CNKI was conducted by the authors to identify eligible publications, and twenty-two publications were finally found to be eligible for merged quantitative analyses.

**Results:**

The merged quantitative analyses revealed that *ADIPOQ* + 45T/G (rs2241766) polymorphism was significantly associated with the risk of GDM in overall population (dominant comparison: OR = 0.70, p < 0.001; recessive comparison: OR = 1.95, p < 0.001; over-dominant comparison: OR = 1.18, p = 0.03; allele comparison: OR = 0.71, p < 0.001) and Asians (dominant comparison: OR = 0.70, p < 0.001; recessive comparison: OR = 1.94, p < 0.001; allele comparison: OR = 0.72, p < 0.001). Nevertheless, we did not observe any positive results for *TNF-α* − 238G/A (rs361525), *TNF-α* − 308G/A (rs1800629), *IL6* − 174G/C (rs1800795), *IL-10* − 819C/T (rs1800871), *IL-10* − 592C/A (rs1800872), *IL-10* − 1082A/G (rs1800896) and *ADIPOQ* + 276G/T (rs1501299) polymorphisms.

**Conclusions:**

The present meta-analysis shows that among investigated *TNF-α*, *IL-6*, *IL-10* or *ADIPOQ* polymorphisms*,* only *ADIPOQ* + 45T/G (rs2241766) polymorphism may affect the risk of GDM.

## Background

Gestational diabetes mellitus (GDM) is a very common disorder of glucose metabolism during pregnancy, and it is alarming that beyond poor glycemic control during pregnancy and potential adverse pregnant outcomes, GDM is also correlated with a significantly higher risk of developing type 2 diabetes mellitus (T2DM) and its associated complications in affected subjects [1, 2]. According to recent epidemiological data, it is estimated that around 1–3% of European pregnancies, and 5–10% of Asian pregnancies are affected by GDM [3].

The etiological factors of GDM remain unclear, but accumulating evidence suggests that disturbance of the immune system is a vital contributing factor to onset and development of GDM, and an abnormal imbalance between Th1 and Th2 cells mediated immune responses has also been documented in patients with GDM [4, 5]. It is well established that cytokines play vital roles in regulating T cell mediated immune responses, and therefore it is believed that gene polymorphisms of cytokines may also influence the risk of GDM [6–8].

Adiponectin (ADIPOQ), an adipocytokine that is predominantly secreted from adipocytes, is critical for regulating energy and material metabolism [9, 10]. In addition to modulate metabolic processes, ADIPOQ also has anti-inflammatory property [11, 12], and several previous observational studies have demonstrated that the plasma level of ADIPOQ is decreased in patients with GDM. So it is speculated that *ADIPOQ* polymorphisms may also impact the risk of GDM.

Over the last decade, investigators all over the world have repeatedly attempted to assess the relationships between polymorphisms in *TNF-α*, *IL-6*, *IL-10* or *ADIPOQ* and the risk of GDM, yet the relationships between these gene polymorphisms and the risk of GDM remain inconclusive. Therefore, in this meta-analysis, we aimed to elucidate the associations between polymorphisms in *TNF-α*, *IL-6*, *IL-10* or *ADIPOQ* and the risk of GDM by merging the results of previous publications.

## Methods

The authors strictly adhere to the PRISMA guideline in study design and implementation [13].

### Literature search and inclusion criteria

A thorough literature searching in Medline, Embase, Wanfang, VIP and CNKI was conducted by the authors with the below terms: (Tumor necrosis factor-α OR TNF-α OR Interleukin-6 OR IL-6 OR Interleukin-10 OR IL-10 OR Adiponectin OR ADIPOQ) AND (polymorphism OR polymorphic OR variation OR variant OR mutant OR mutation OR SNP OR genotypic OR genotype OR allelic OR allele) AND (Gestational diabetes mellitus OR GDM). Moreover, we also manually screened the reference lists of retrieved publications to make up for the potential incompleteness of electronic literature searching.

Selection criteria of eligible publications include the following four points: 1. Studies of case–control or cohort design; 2. Explore relationships between polymorphisms in *TNF-α*, *IL-6*, *IL-10* or *ADIPOQ* and the risk of GDM; 3. Give genotypic frequencies of *TNF-α*, *IL-6*, *IL-10* or *ADIPOQ* polymorphisms in cases with GDM and population-based controls; 4. The full manuscript with required genotypic frequencies of *TNF-α*, *IL-6*, *IL-10* or *ADIPOQ* polymorphisms is retrievable or buyable. Articles would be excluded if one of the following three criteria is met: 1. Studies without complete data about genotypic frequencies of *TNF-α*, *IL-6*, *IL-10* or *ADIPOQ* polymorphisms in cases with GDM and population-based controls; 2. Narrative or systematic reviews, meta-analysis or comments; 3. Case series of subjects with GDM only. If duplicate publications are retrieved from literature search, we would only include the most complete one for quantitative analyses.

### Data extraction and quality assessment

The authors extracted the following data items from eligible publications: 1. Last name of the first author; 2. Publication year; 3. Country and ethnicity of study subjects; 4. The number of cases with GDM and population-based controls; 5. Genotypic frequencies of *TNF-α*, *IL-6*, *IL-10* or *ADIPOQ* polymorphisms in cases with GDM and population-based controls. Hardy–Weinberg equilibrium was then tested by using genotypic frequencies of *TNF-α*, *IL-6*, *IL-10* or *ADIPOQ* polymorphisms. The quality of eligible publications was assessed by the Newcastle–Ottawa scale (NOS) [14], and these with a score of 7–9 were considered to be publications of good quality. The NOS assess the quality of eligible studies from three aspects: selection of cases and controls [adequate definition of cases (one point); representativeness of the cases (one point); population-based controls (one point); controls do not have history of GDM (one point)], comparability of cases and controls [ethnicity (one point); age (one point)] and exposure in cases and controls [ascertainment of exposure (one point); same method of ascertainment for cases and controls (one point); same non-response rate between cases and controls (one point)]. Two authors extracted data and assessed quality of eligible publications in parallel. When necessary, the reviewers would write to the corresponding authors of eligible studies for extra information or raw data. A thorough discussion until a consensus is reached would be endorsed in case of any discrepancy between two authors.

### Statistical analyses

All statistical analyses were performed with the Cochrane Review Manager software version 5.3.3 (The Cochrane Collaboration, Software Update, Oxford, United Kingdom). Relationships between *TNF-α*, *IL-6*, *IL-10* or *ADIPOQ* polymorphisms and the risk of GDM were estimated by using odds ratio and its 95% confidence interval (chi-square test). The statistically significant p value was set at 0.05. All investigated polymorphisms contain a major allele (M) and a minor allele (m), the dominant comparison was defined as MM vs. Mm + mm, the recessive comparison was defined as mm vs. MM + Mm, the over-dominant comparison was defined as Mm vs. MM + mm, and the allele comparison was defined as M vs. m (MM stands for homozygote of the major allele, Mm stands for heterozygote of the major allele and the minor allele, and mm stands for homozygote of the minor allele). The authors used I^2^ statistics to assess whether significant heterogeneities existed among eligible publications. The authors would use DerSimonian–Laird method, which is also known as the random effect model, to merge the results of eligible publications if I^2^ is larger than 50%. Otherwise, the authors would use Mantel–Haenszel method, which is also known as the fixed effect model, to merge the results of eligible publications. Meanwhile, subgroup analyses by ethnic groups were also conducted by the authors. Stabilities of quantitative analyses results were tested by deleting one eligible publication each time, and then merging the results of the rest of eligible publications. Publication biases were evaluated by assessing symmetry of funnel plots.

## Results

### Characteristics of included studies

**Fig. 1:**
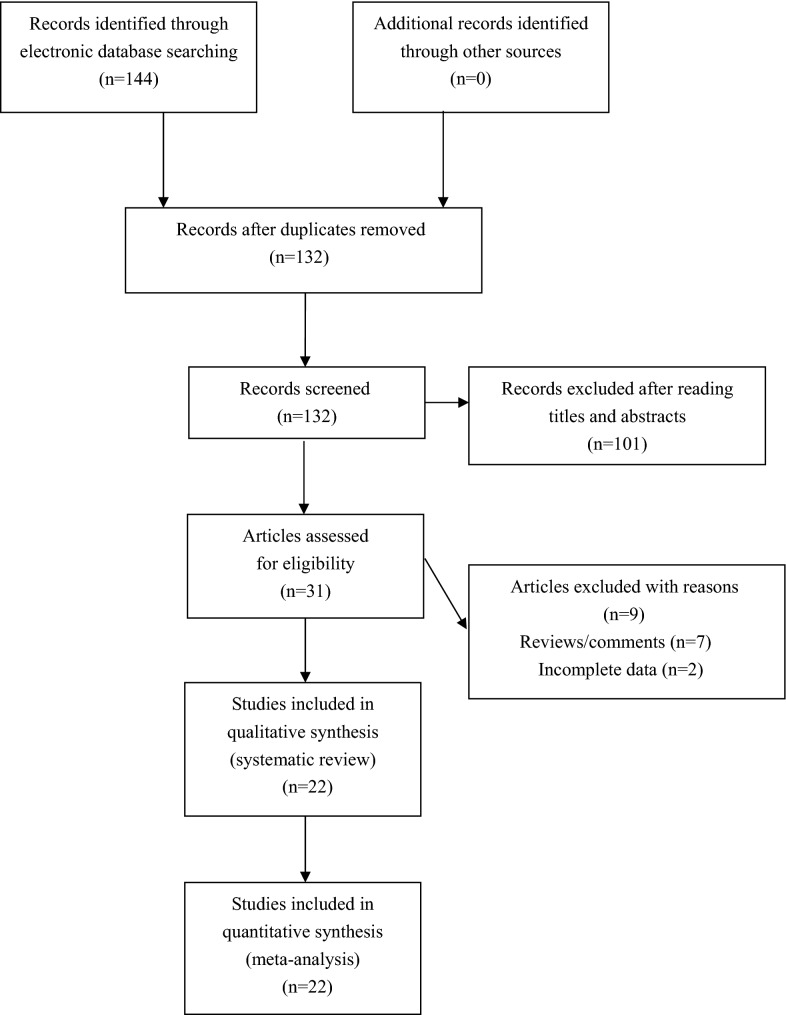
Flowchart of study selection for this meta-analysis

One hundred and forty-four publications were retrieved by the authors by using our searching strategy. Thirty-one publications were selected to screen for eligibility after omitting unrelated and repeated publications. Seven reviews were then excluded, and another two publications without all necessary genotypic data were further excluded by the authors. Totally twenty-two publications met the selection criteria, and were finally included for quantitative analyses (Fig. 1). Data extracted from eligible publications were summarized in Table 1.

**Table 1:** The characteristics of included studies in current meta-analysis

First author, year	Country	Ethnicity	Sample size	Genotypes (wtwt/wtmt/mtmt)			p-value for HWE	NOS score
				Cases	Controls			
TNF-α − 238 G/A rs361525								
Guzmán-Flores 2013	Mexico	Mixed	51/44	41/9/1		37/7/0	0.566	7
Yang 2005	China	Asian	120/120	107/13/0		109/11/0	0.599	7
TNF-α − 308 G/A rs1800629								
Feng 2019	China	Asian	105/84	94/11/0		78/6/0	0.734	8
Gueuvoghlanian-Silva 2012	Brazil	Mixed	79/168	59/18/2		133/31/4	0.192	7
Guzmán-Flores 2013	Mexico	Mixed	51/44	43/7/1		39/5/0	0.689	7
Jing 2015	China	Asian	124/65	103/14/7		51/11/3	0.039	7
Montazeri 2010	Malaysia	Asian	110/102	103/4/3		94/6/2	< 0.001	8
Wang 2016	China	Asian	50/100	26/14/10		51/38/11	0.341	7
Yang 2005	China	Asian	120/120	91/29/0		106/14/0	0.497	7
IL6 − 174 G/C rs1800795								
Feng 2019	China	Asian	50/45	48/2/0		42/3/0	0.817	8
Gueuvoghlanian-Silva 2012	Brazil	Mixed	79/165	47/24/8		104/52/9	0.463	7
Jing 2018	China	Asian	124/65	112/11/1		63/2/0	0.900	7
IL-10 − 819C/T rs1800871								
Kang 2019	Taiwan	Asian	72/100	33/32/7		49/41/10	0.742	8
Montazeri 2010	Malaysia	Asian	110/102	38/58/14		37/46/19	0.486	8
IL-10 − 592C/A rs1800872								
Kang 2019	Taiwan	Asian	72/100	33/32/7		51/39/10	0.533	8
Majcher 2019	Poland	Caucasian	204/207	124/68/12		115/71/21	0.051	8
Montazeri 2010	Malaysia	Asian	110/102	44/50/16		30/58/14	0.094	8
IL-10 − 1082A/G rs1800896								
Gueuvoghlanian-Silva 2012	Brazil	Mixed	80/165	43/29/8		84/66/15	0.700	7
Kang 2019	Taiwan	Asian	72/100	64/8/0		88/12/0	0.523	8
Montazeri 2010	Malaysia	Asian	110/102	81/24/5		74/24/4	0.265	8
ADIPOQ + 45T/G rs2241766								
Daher 2011	Brazil	Mixed	79/169	61/15/3		134/32/3	0.505	7
Feng 2019	China	Asian	135/135	53/63/19		70/55/10	0.858	8
Gao 2016	China	Asian	150/150	59/66/25		81/57/12	0.659	8
Han 2012	China	Asian	152/120	63/71/18		64/50/6	0.339	8
Li 2013	China	Asian	264/172	134/113/17		97/66/9	0.604	8
Li 2017	China	Asian	130/130	53/63/14		63/60/7	0.128	8
Low 2011	Malaysia	Asian	26/53	11/13/2		35/17/1	0.512	7
Luan 2015	China	Asian	60/60	33/21/6		29/26/5	0.806	7
Luo 2019	China	Asian	150/150	70/66/14		75/67/8	0.155	7
Takhshid 2015	Iran	Mixed	65/70	37/28/0		54/16/0	0.280	7
Zhang 2014	China	Asian	98/135	38/43/17		73/51/11	0.622	8
Zheng 2012	China	Asian	152/248	63/71/18		116/114/18	0.159	7
ADIPOQ + 276G/T rs1501299								
Gao 2016	China	Asian	150/150	66/69/15		75/60/15	0.560	8
Han 2012	China	Asian	152/120	74/66/12		56/53/11	0.760	8
Li 2017	China	Asian	130/130	64/58/8		60/56/14	0.863	8
Luan 2015	China	Asian	60/60	27/26/7		32/25/3	0.499	7
Luo 2019	China	Asian	160/150	90/52/8		84/55/11	0.632	7
Zhang 2014	China	Asian	98/135	43/45/10		68/54/13	0.636	8
Zheng 2012	China	Asian	152/248	74/66/12		121/103/24	0.761	7

wt, wild type; mt, mutant type; HWE, Hardy–Weinberg equilibrium; NOS, Newcastle–Ottawa scale; NA, not available

### Quantitative analyses of investigated polymorphisms and the risk of GDM

Seven publications assessed relationship between *TNF-α* polymorphisms and the risk of GDM, three publications assessed relationship between *IL-6* polymorphisms and the risk of GDM, four publications assessed relationship between *IL-10* polymorphisms and the risk of GDM, and twelve publications assessed relationship between *ADIPOQ* polymorphisms and the risk of GDM. The merged quantitative analyses revealed that *ADIPOQ* + 45T/G (rs2241766) polymorphism was significantly associated with the risk of GDM in overall population (dominant comparison: OR = 0.70, p < 0.001; recessive comparison: OR = 1.95, p < 0.001; over-dominant comparison: OR = 1.18, p = 0.03; allele comparison: OR = 0.71, p < 0.001) and Asians (dominant comparison: OR = 0.70, p < 0.001; recessive comparison: OR = 1.94, p < 0.001; allele comparison: OR = 0.72, p < 0.001). Nevertheless, we did not observe any positive results for *TNF-α* − 238G/A (rs361525), *TNF-α* − 308G/A (rs1800629), *IL6* − 174G/C (rs1800795), *IL-10* − 819C/T (rs1800871), *IL-10* − 592C/A (rs1800872), *IL-10* − 1082A/G (rs1800896) and *ADIPOQ* + 276G/T (rs1501299) polymorphisms (see Table 2 and Additional file 1: Figure S1).

**Table 2:** Merged quantitative analyses results of the current study

Variables	Sample size	Dominant comparison (MM vs. Mm + mm)		Recessive comparison (mm vs. MM + Mm)		Overdominant comparison (Mm vs. MM + mm)		Allele comparison (M vs. m)	
		*p* value	OR (95% CI)	*p* value	OR (95% CI)	*p* value	OR (95% CI)	*p* value	OR (95% CI)
TNF-α − 238 G/A rs361525									
Overall (Mixed population)	171/164	0.53	0.81 (0.42–1.57)	0.55	2.64 (0.11–66.55)	0.63	1.18 (0.60–2.29)	0.45	0.79 (0.42–1.48)
TNF-α − 308 G/A rs1800629									
Overall (Mixed population)	639/683	0.15	0.81 (0.60–1.08)	0.16	1.60 (0.84–3.04)	0.42	1.14 (0.83–1.55)	0.09	0.80 (0.62–1.04)
Asian	509/471	0.29	0.83 (0.58–1.17)	0.17	1.66 (0.81–3.41)	0.97	1.01 (0.54–1.89)	0.18	0.81 (0.60–1.10)
IL6 − 174 G/C rs1800795									
Overall (Mixed population)	253/275	0.32	0.78 (0.48–1.27)	0.18	1.91 (0.74–4.96)	0.75	1.09 (0.65–1.80)	0.15	0.74 (0.49–1.11)
Asian	174/110	0.28	0.55 (0.19–1.62)	0.78	1.59 (0.06–39.61)	0.35	1.68 (0.57–4.97)	0.23	0.52 (0.18–1.50)
IL-10 − 819C/T rs1800871									
Overall (Asian)	182/202	0.64	0.91 (0.60–1.37)	0.33	0.74 (0.40–1.35)	0.26	1.26 (0.84–1.89)	0.88	1.02 (0.76–1.38)
IL-10 − 592C/A rs1800872									
Overall (Mixed population)	386/409	0.20	1.20 (0.91–1.60)	0.34	0.79 (0.50–1.27)	0.49	0.90 (0.68–1.20)	0.23	1.14 (0.92–1.42)
Asian	182/202	0.47	1.16 (0.77–1.76)	0.92	1.03 (0.56–1.91)	0.44	0.85 (0.57–1.28)	0.63	1.07 (0.80–1.45)
IL-10 − 1082A/G rs1800896									
Overall (Mixed population)	262/367	0.64	1.09 (0.75–1.58)	0.75	1.13 (0.53–2.39)	0.52	0.88 (0.60–1.29)	0.79	1.04 (0.77–1.42)
Asian	182/202	0.80	1.07 (0.64–1.78)	0.82	1.17 (0.30–4.47)	0.73	0.91 (0.53–1.55)	0.89	1.03 (0.66–1.63)
ADIPOQ + 45T/G rs2241766									
Overall (Mixed population)	1461/1592	*< 0.001*	*0.70 (0.60–0.81)*	*< 0.001*	*1.95 (1.48–2.56)*	*0.03*	*1.18 (1.02–1.37)*	* < 0.001*	*0.71 (0.64–0.80)*
Asian	1317/1353	*< 0.001*	*0.70 (0.60–0.82)*	*< 0.001*	*1.94 (1.47–2.57)*	0.08	1.15 (0.98–1.34)	* < 0.001*	*0.72 (0.64–0.81)*
ADIPOQ + 276G/T rs1501299									
Overall (Asian)	902/993	0.50	0.94 (0.78–1.13)	0.41	0.87 (0.63–1.21) 0.87 (0.63–1.21)	0.49	1.07 (0.89–1.28)	0.61	0.96 (0.84–1.11)

All investigated polymorphisms contain a major allele (M) and a minor allele (m), The dominant comparison was defined as MM vs. Mm + mm, the recessive comparison was defined as mm vs. MM + Mm, the over-dominant comparison was defined as Mm vs. MM + mm, and the allele comparison was defined as M vs. m (MM stands for homozygote of the major allele, Mm stands for heterozygote of the major allele and the minor allele, and mm stands for homozygote of the minor allele)

The values in italics represent there is statistically significant differences between cases and controls

OR, odds ratio; CI, confidence interval; NA, not available; UC, ulcerative colitis; CD, Crohn’s disease

### Sensitivity analyses

The authors examined stabilities of quantitative analyses results by deleting one eligible publication each time, and then merging the results of the rest of publications. The trends of associations were not significantly altered in sensitivity analyses, which indicated that from statistical perspective, our quantitative analyses results were reliable and stable.

### Publication biases

The authors examined potential publication biases in this meta-analysis by assessing symmetry of funnel plots. Funnel plots were found to be generally symmetrical, which indicated that our merged quantitative analyses results were not likely to be seriously deteriorated by publication biases (see Additional file 2: Figure S2).

## Discussion

This is so far the first meta-analysis regarding *TNF-α*, *IL-6* or *IL-10* polymorphisms and the risk of GDM, and it is also so far the most complete meta-analysis regarding *ADIPOQ* polymorphisms and the risk of GDM. The quantitative analyses results demonstrated that *ADIPOQ* + 45T/G (rs2241766) polymorphism was significantly associated with the risk of GDM in overall population and Asians. However, we did not observe any positive results for *TNF-α* − 238 G/A (rs361525), *TNF-α* − 308 G/A (rs1800629), *IL6* − 174 G/C (rs1800795), *IL-10* − 819C/T (rs1800871), *IL-10* − 592C/A (rs1800872), *IL-10* − 1082A/G (rs1800896) and *ADIPOQ* + 276G/T (rs1501299) polymorphisms (Genomic position, reference genome used, minor allele frequency and functional consequence of investigated polymorphisms can be obtained at https://www.ncbi.nlm.nih.gov/snp using the SNP ID numbers). It is worth noting that the pooled analyses for the *ADIPOQ* + 45T/G (rs2241766) polymorphism were based on over 3000 study subjects, and no obvious heterogeneity among eligible studies was detected, so this positive finding was quite statistically robust.

There are a few points that should be considered when interpreting our findings. First, based on findings of previous observational studies, it is believed that investigated *TNF-α*, *IL-6*, *IL-10* and *ADIPOQ* polymorphisms may alter transcription activity of *TNF-α*, *IL-6*, *IL-10* and *ADIPOQ*, and this is also the primary reason why these polymorphisms have been repeatedly analyzed with regard to the risk of different types of diseases including GDM [15–17]. Nevertheless, we have to point out that the functionalities of investigated polymorphisms remain uncertain, and thus the exact mechanisms underlying the observed association between *ADIPOQ* + 45T/G (rs2241766) polymorphism and the risk of GDM still require further explorations. Second, despite that our quantitative analyses were derived from integrating the results of all published studies. We should admit that the sample sizes of many comparisons were still relatively small, and thus may be still inadequate to detect the real associations between investigated polymorphisms and the risk of GDM. So further genetic association studies with larger sample sizes in other populations or ethnicities are still warranted to confirm our findings. Third, we also wish to study polymorphic loci of other cytokines in this meta-analysis. Nevertheless, our initial literature searching did not reveal sufficient eligible publications to support quantitative analyses for any polymorphic loci of other cytokines, which include *IL-1*, *IL-2*, *IL-4*, *IL-8*, *IL-12* and *IL-18*, so we only explored associations with the risk of GDM for *TNF-α*, *IL-6* and *IL-10* polymorphisms in our quantitative analyses. Fourth, although a recent meta-analysis by Huang et al. also tried to elucidate the associations between *ADIPOQ* polymorphisms and GDM [18], it should be noted that compared to the previous work, the overall pooled sample size of our quantitative analyses was around one thousand larger. Taken into account that similar positive findings were documented in these two meta-analyses, we believe that the current meta-analysis serves as a valuable confirmation to pre-existing literatures. Fifth, for a single genetic association study, especially a genome wide association study (GWAS), in which many gene polymorphisms were explored in a group of study subjects at the same time, Bonferroni-correction should be conducted since multiple tests were performed simultaneously. Considering that the investigated polymorphisms may somehow be connected with each other, the possibility of getting false positive results (type I error) would for sure significantly increase when many gene polymorphisms are studied in a group of study subjects at the same time, and this is also the reason why in a GWAS, the p values should be generally set at a much lower level to avoid potential type I error. However, in this meta-analysis, although multiple polymorphisms were analyzed, since different studies for enrolled for different gene polymorphisms, the study subjects of each polymorphism were actually different, and so the status of this meta-analysis is totally different from a single GWAS in which many gene polymorphisms were studied in the exact same population. If we use Bonferroni-correction in a meta-analysis, the possibility of getting false negative results (type II error) would certainly increase to an unbearable high level, so Bonferroni-correction was not performed. Besides, the p values of dominant, recessive and allele comparisons for *ADIPOQ* + 45T/G (rs2241766) polymorphism were all lower than 0.001, so even if we set the significance threshold at a lower level such as 0.00625 (0.05/8 since eight polymorphisms were analyzed in this meta-analysis), the positive results obtained in this meta-analysis still won't be altered. Sixth, no GWAS reports were found to be eligible for inclusion in this meta-analysis since the authors would usually only provide allelic distributions of investigated polymorphisms, but not detailed genotypic distributions in GWAS reports. In our meta-analysis, four different genetic models were compared for each polymorphism so as to more comprehensively assess the relationships between investigated polymorphisms and the risk of GDM. So if detailed genotypic distribution data could not be obtained from a certain study, we would not include it for pooled analyses even if it is a GWAS. Seventh, it is worth noting that previous meta-analyses found that *IL-10* − 819C/T (rs1800871), *IL-10* − 592C/A (rs1800872), *IL-10* − 1082A/G (rs1800896), *TNF-α* − 308 G/A (rs1800629) and *ADIPOQ* + *45T/G* (rs2241766) were significantly associated with the risk of T2DM, whereas *IL6* − 174 G/C (rs1800795) was significantly associated with the risk of nephrology in T2DM patients [19–22]. Considering that GDM patients have a significantly higher risk of developing T2DM and its associated complications, it is believed that GDM and T2DM may share similar genetic traits. In our meta-analysis, only *ADIPOQ* + *45T/G* (rs2241766) polymorphism was found to be associated with the risk of GDM. Nevertheless, since our pooled analyses for *TNF-α*, *IL-6* and *IL-10* polymorphisms were only based on limited number of studies, future studies with larger sample sizes are still warranted to test our findings.

The major limitations of this meta-analysis were summarized as below. Firstly, we need to admit that our quantitative analyses results were unadjusted. Without access to raw data of eligible publications, we can only estimate associations based on re-calculations of raw genotypic frequencies, so it should be acknowledged that lack of further adjustment for baseline characteristics may certainly influence authenticity of our findings [23]. Secondly, environmental factors may also affect relationships between *TNF-α*, *IL-6*, *IL-10* or *ADIPOQ* polymorphisms and the risk of GDM. However, the majority of authors only paid attention to genetic analyses in their publications, so it is impossible for us to explore genetic-environmental interactions in a secondary analysis of previous publications [24]. Thirdly, we did not enroll 'grey literatures' (Grey literatures refer to datasets or reports that are produced by all levels of government, academics or business institutions, but are not formally published in peer-reviewed scientific journals) for quantitative analyses because it is almost impossible for us to extract all required data items from these literatures or throughly assess their quality using the NOS scale. Nevertheless, since we did not include grey literatures for quantitative analyses, despite that funnel plots were found to be in general symmetrical, we admitted that publication biases still may impact reliability of our quantitative analyses results [25].

## Conclusion

In conclusion, this meta-analysis demonstrates that among investigated *TNF-α*, *IL-6*, *IL-10* or *ADIPOQ* polymorphisms*,* only *ADIPOQ* + 45T/G (rs2241766) polymorphism may affect the risk of GDM. However, further studies with larger sample sizes are still needed to confirm our findings. Besides, scholars should also try to explore the exact underlying molecular mechanisms of the observed association between *ADIPOQ* + 45T/G (rs2241766) polymorphism and GDM.

## Supplementary information

**Additional file 1.** Forest plots of investigated polymorphisms.

**Additional file 2.** Funnel plots of investigated polymorphisms.

## Data Availability

Not applicable.

## Abbreviations

GDM: gestational diabetes mellitus
TNF-α: tumor necrosis factor-α
IL-6: interleukin-6
IL-10: interleukin-10
ADIPOQ: adiponectin
HWE: Hardy–Weinberg equilibrium
NOS: Newcastle–Ottawa scale
OR: odds ratios
CI: confidence intervals
