# Supplementary material for: Whether the risk of gestational diabetes mellitus is affected by TNF-α, IL-6, IL-10 or ADIPOQ polymorphisms: a meta-analysis
Source: Diabetol Metab Syndr. 2020 Sep 17;12:81. doi: 10.1186/s13098-020-00582-8 (PMC7499992; doi:10.1186/s13098-020-00582-8)
Supplement: Supplementary file 1 — Additional file 1. Forest plots of investigated polymorphisms. [file 13098_2020_582_MOESM1_ESM.docx]

**Supplementary Figure 1. Forest plots of investigated polymorphisms**


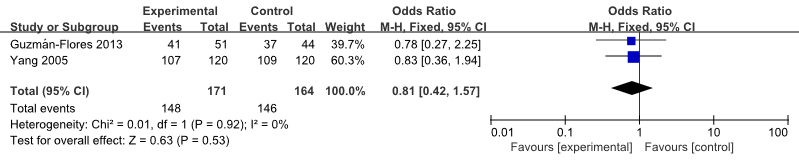


Forest plot of TNF-α -238 G/A polymorphism and GDM under dominant comparison


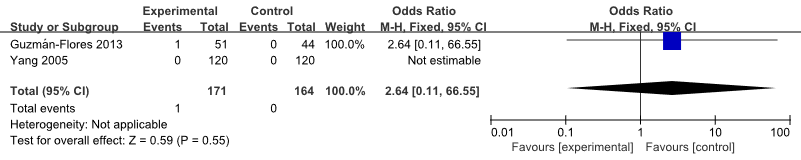


Forest plot of TNF-α -238 G/A polymorphism and GDM under recessive comparison


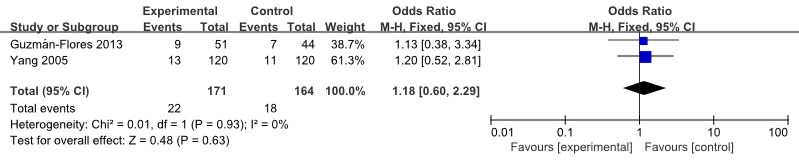


Forest plot of TNF-α -238 G/A polymorphism and GDM under over-dominant comparison


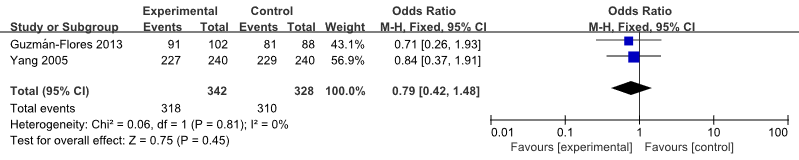


Forest plot of TNF-α -238 G/A polymorphism and GDM under allele comparison


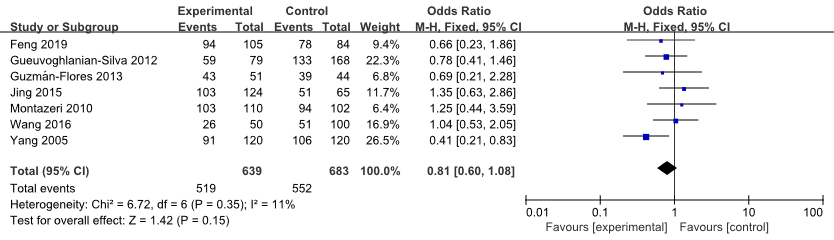


Forest plot of TNF-α -308 G/A polymorphism and GDM under dominant comparison


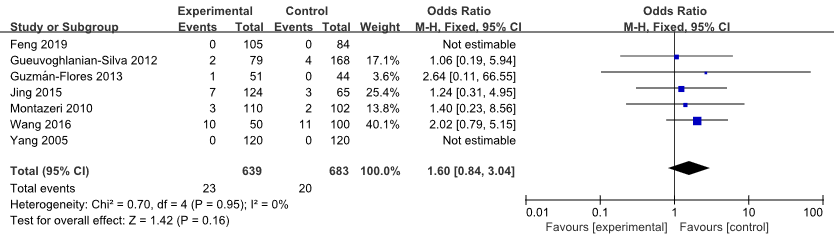


Forest plot of TNF-α -308 G/A polymorphism and GDM under recessive comparison


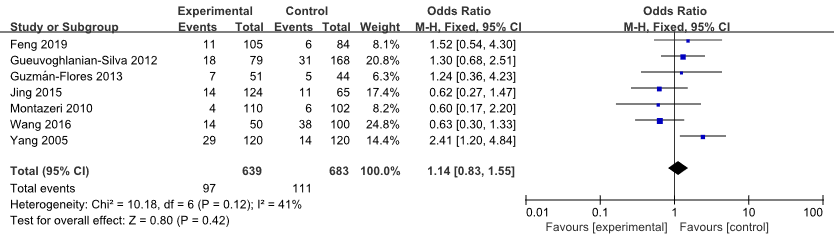


Forest plot of TNF-α -308 G/A polymorphism and GDM under over-dominant comparison


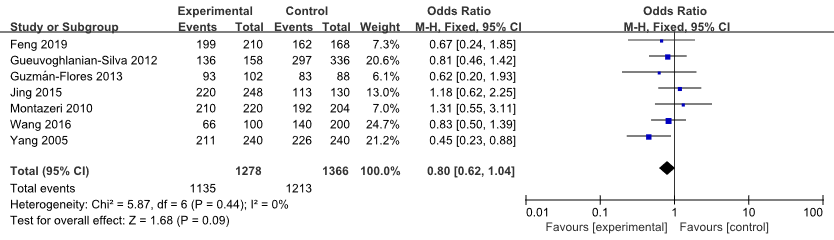


Forest plot of TNF-α -308 G/A polymorphism and GDM under allele comparison


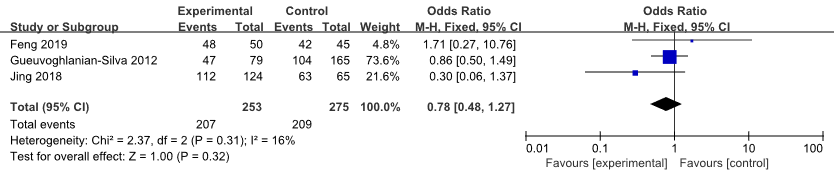


Forest plot of IL6 -174 G/C polymorphism and GDM under dominant comparison


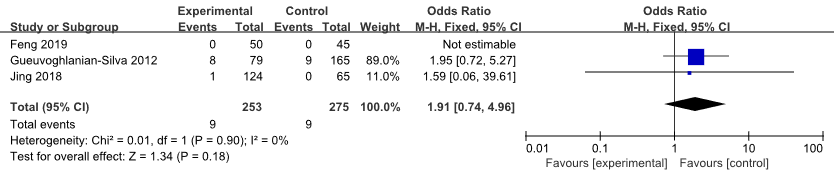


Forest plot of IL6 -174 G/C polymorphism and GDM under recessive comparison


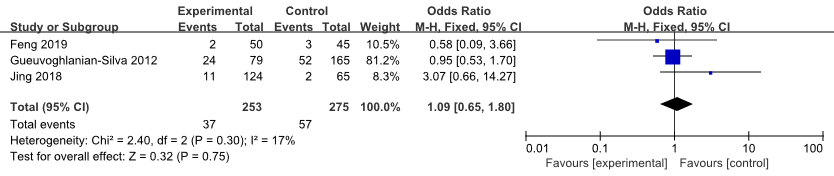


Forest plot of IL6 -174 G/C polymorphism and GDM under over-dominant comparison


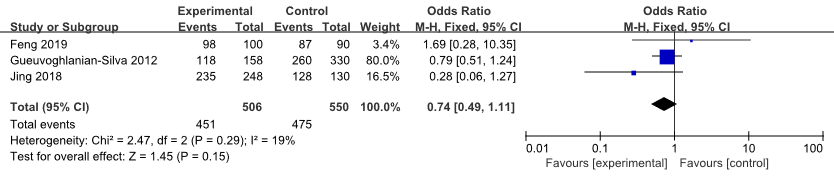


Forest plot of IL6 -174 G/C polymorphism and GDM under allele comparison


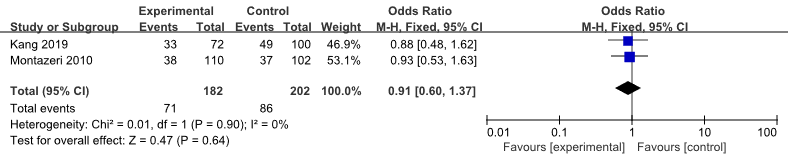


Forest plot of IL-10 -819C/T polymorphism and GDM under dominant comparison


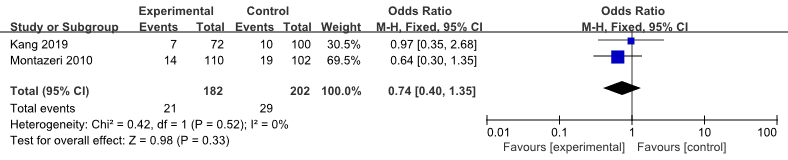


Forest plot of IL-10 -819C/T polymorphism and GDM under recessive comparison


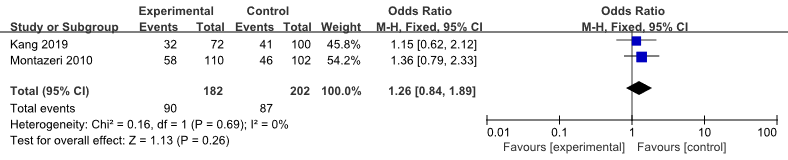


Forest plot of IL-10 -819C/T polymorphism and GDM under over-dominant comparison


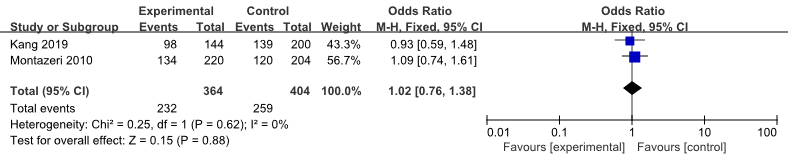


Forest plot of IL-10 -819C/T polymorphism and GDM under allele comparison


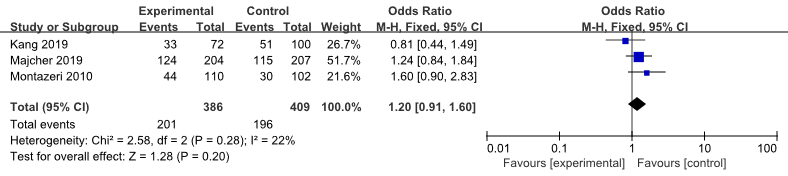


Forest plot of IL-10 -592C/A polymorphism and GDM under dominant comparison


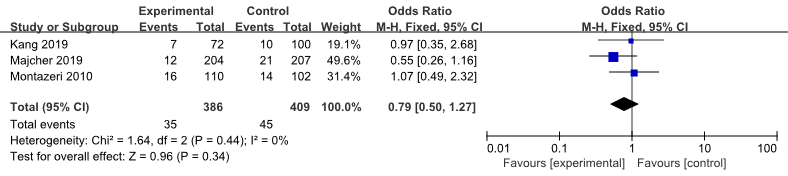


Forest plot of IL-10 -592C/A polymorphism and GDM under recessive comparison


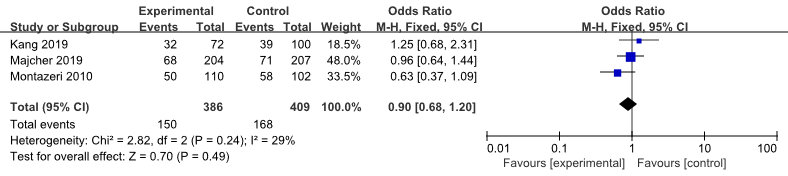


Forest plot of IL-10 -592C/A polymorphism and GDM under over-dominant comparison


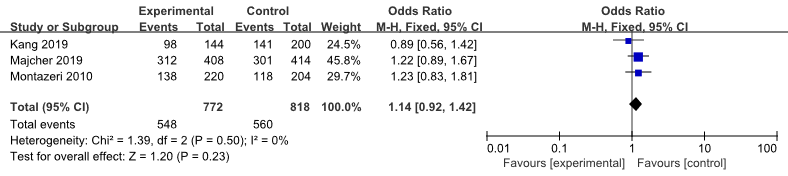


Forest plot of IL-10 -592C/A polymorphism and GDM under allele comparison


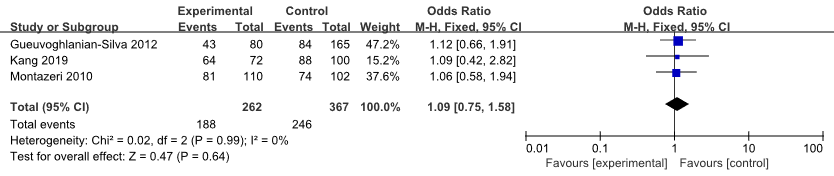


Forest plot of IL-10 -1082A/G polymorphism and GDM under dominant comparison


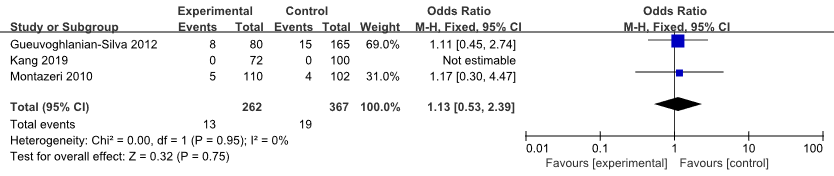


Forest plot of IL-10 -1082A/G polymorphism and GDM under recessive comparison


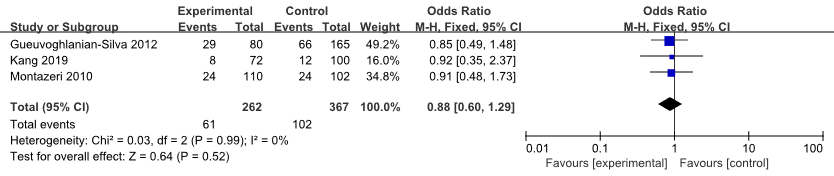


Forest plot of IL-10 -1082A/G polymorphism and GDM under over-dominant comparison


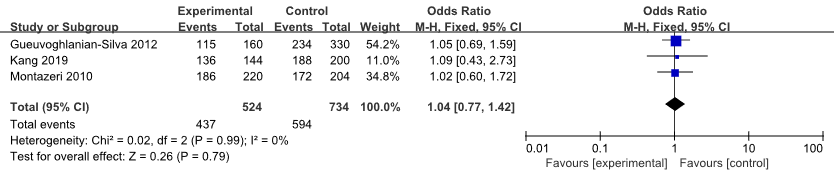


Forest plot of IL-10 -1082A/G polymorphism and GDM under allele comparison


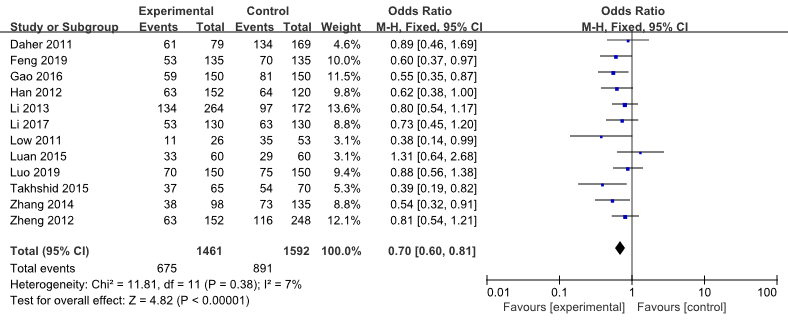


Forest plot of ADIPOQ +45T/G polymorphism and GDM under dominant comparison


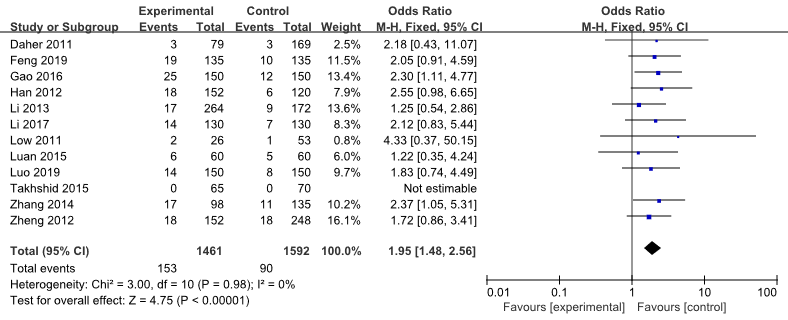


Forest plot of ADIPOQ +45T/G polymorphism and GDM under recessive comparison


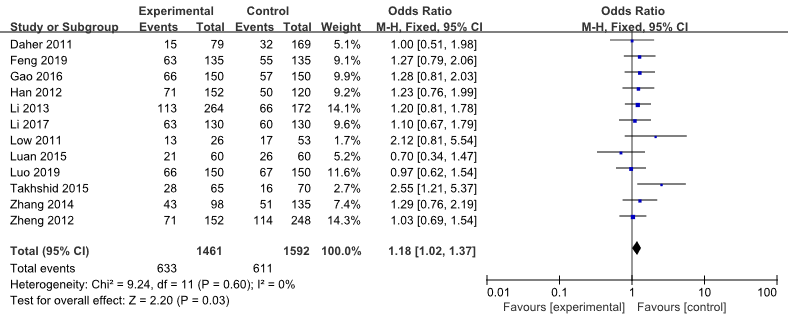


Forest plot of ADIPOQ +45T/G polymorphism and GDM under over-dominant comparison


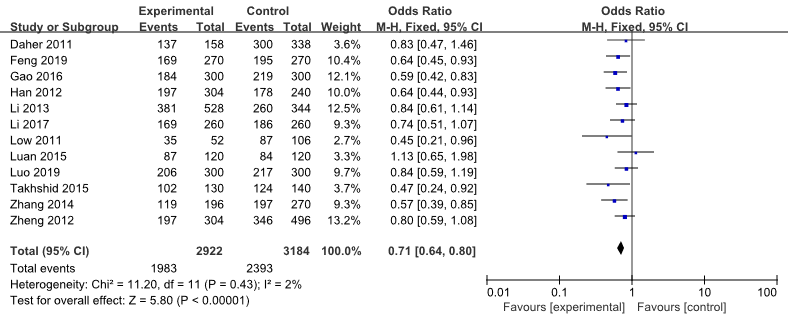


Forest plot of ADIPOQ +45T/G polymorphism and GDM under allele comparison


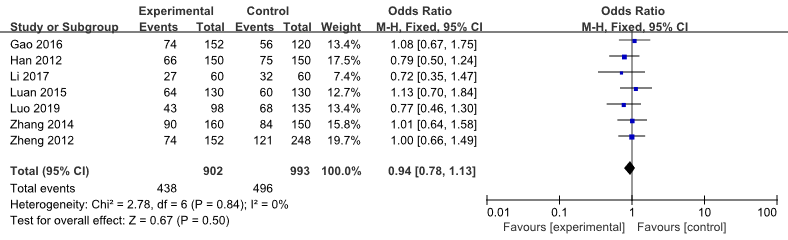


Forest plot of ADIPOQ +276G/T polymorphism and GDM under dominant comparison


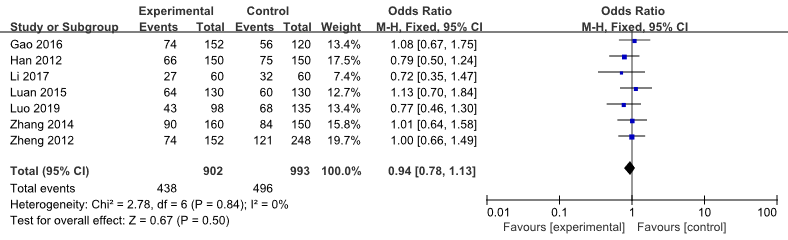


Forest plot of ADIPOQ +276G/T polymorphism and GDM under recessive comparison


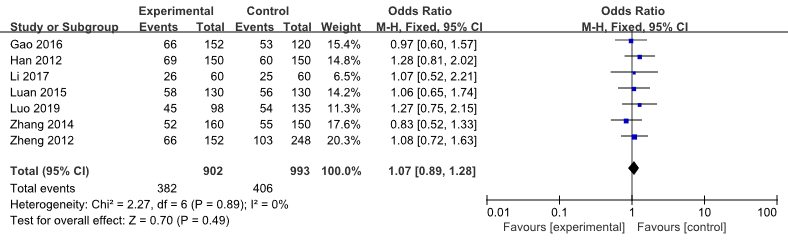


Forest plot of ADIPOQ +276G/T polymorphism and GDM under over-dominant comparison


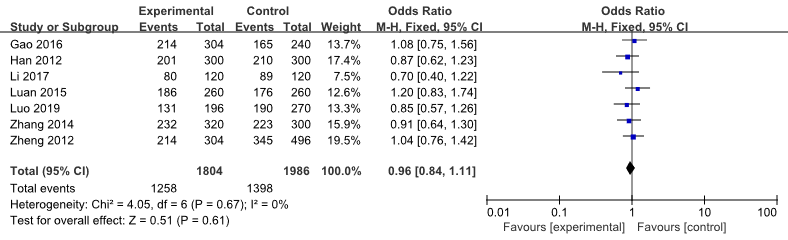


Forest plot of ADIPOQ +276G/T polymorphism and GDM under allele comparison
